# Ancient hybridization and mtDNA introgression behind current paternal leakage and heteroplasmy in hybrid zones

**DOI:** 10.1038/s41598-019-55764-w

**Published:** 2019-12-16

**Authors:** Valentina Mastrantonio, Sandra Urbanelli, Daniele Porretta

**Affiliations:** Department of Environmental Biology, Sapienza University of Rome, Rome, Italy

**Keywords:** Evolutionary ecology, Evolution

## Abstract

Hybridization between heterospecific individuals has been documented as playing a direct role in promoting paternal leakage and mitochondrial heteroplasmy in both natural populations and laboratory conditions, by relaxing the egg-sperm recognition mechanisms. Here, we tested the hypothesis that hybridization can lead to mtDNA heteroplasmy also indirectly via mtDNA introgression. By using a phylogenetic approach, we showed in two reproductively isolated beetle species, *Ochthebius quadricollis* and *O. urbanelliae*, that past mtDNA introgression occurred between them in sympatric populations. Then, by developing a multiplex allele-specific PCR assay, we showed the presence of heteroplasmic individuals and argue that their origin was through paternal leakage following mating between mtDNA-introgressed and pure conspecific individuals. Our results highlight that mtDNA introgression can contribute to promote paternal leakage, generating genetic novelty in a way that has been overlooked to date. Furthermore, they highlight that the frequency and distribution of mtDNA heteroplasmy can be deeply underestimated in natural populations, as *i*) the commonly used PCR-Sanger sequencing approach can fail to detect mitochondrial heteroplasmy, and *ii*) specific studies aimed at searching for it in populations where mtDNA-introgressed and pure individuals co-occur remain scarce, despite the fact that mtDNA introgression has been widely documented in several taxa and populations.

## Introduction

Mitochondrial DNA (mtDNA) has been one of the most popular genetic markers for studies concerning species ecology and evolution. In animals, mtDNA has been widely used to investigate population demography, to reconstruct species phylogenies and phylogeography, and, more recently, it is even being used to identify cryptic genetic diversity and classify living organisms^1–4^.

Strict maternal inheritance has been undoubtedly one of the most important features underlying the mtDNA success^5–8^. As a consequence of this way of inheritance, a condition of homoplasmy is set up in the offspring and maintained across generations. Different mechanisms during gametogenesis and after fertilization have been described, which ensure offspring homoplasmy by selectively destroying sperm mitochondria^8–10^. In mammals, after fertilization, paternal mitochondria are ubiquitinated and then degraded by enzymes^11^; in the rice fish *Oryzias latipes*, paternal mtDNA is degraded^12^; in the nematode *Caenorhabditis elegans* and the fruit fly *Drosophila melanogaster*, paternal mitochondria are destroyed by autophagy^9,13^. Basically, the above selective mechanisms are based on the egg-sperm recognition, where a factor coded by the nuclear genome occurring in the eggs is able to recognize a signal produced by the paternal genome located in the sperm mitochondria, promoting their destruction just after fertilization^8^. However, increasing evidence is showing that maternal inheritance is often not strict, but that male mtDNA transmission can occur (a process termed paternal leakage), leading to a condition of mitochondrial heteroplasmy, where mitochondrial genomes of both parents occur within an individual (paternal leakage-driven heteroplasmy)^8,10,14^.

In recent years, hybridization has been shown to play a direct role in promoting paternal leakage and heteroplasmy. Indeed, given that the efficiency of egg-sperm recognition mechanisms decreases when genetic divergence between parents increases, hybridization between individuals belonging to genetically divergent lineages favours paternal leakage^8^. Documented cases have been observed in a wide range of vertebrate and invertebrate taxa, including insects, birds, amphibians and mammals, and most of them have been indeed shown to involve hybrid individuals from experimental crosses or natural hybrid zones^8,10,15–19^.

In this paper, we argue that hybridization can promote mtDNA paternal leakage and heteroplasmy also indirectly, via mtDNA introgression. Following hybridization, mtDNA introgression, defined as the permanent incorporation of genes from one species into another, has been frequently reported to such an extent that complete mtDNA replacement can also occur^20–26^. In many cases, however, mtDNA-introgressed and pure conspecific individuals co-exist. For example, mtDNA introgression has been observed between the sea-rock pool mosquitoes *Aedes mariae* and *Ae. zammitii* in a recently established hybrid zones along the Italian coasts of the Adriatic Sea (e.g. as a consequence of human-mediated introduction). After its introduction, *Ae. mariae* diffused along a transect of about 20 km, coexisting in syntopy with *Ae. zammitii*. Reproductive isolation between the two species is not completed and ongoing hybridization occurs which led to bidirectional mtDNA introgression (mtDNA-introgressed individuals reached 25% and 14% in *Ae. mariae* and *Ae. zammitii*, respectively)^25^.

The co-occurrence of mtDNA-introgressed and pure conspecific individuals has been observed not only when hybridization is ongoing, but also when ancient introgression occurred between taxa that are currently reproductively isolated^27–29^. For example, ancient introgression following hybridization has been documented in a zone of parapatry between the two European treefrogs *Hyla arborea* and *H. intermedia*^27^. Genetic analyses using both nuclear and mitochondrial diagnostic markers showed the lack of current gene exchange between the two species, while introgressed alleles were observed in both species and in all markers analysed. In particular, mtDNA of *H. arborea* was observed in three individuals of *H. intermedia*^27^.

Here, we suggest that in populations where conspecific mtDNA-introgressed and pure conspecific individuals co-exist, a mating between pure and introgressed individuals could favour paternal leakage, by relaxing the egg-sperm recognition mechanisms that prevent it, like a mating between two heterospecific individuals. If so, it can be expected that paternal leakage-driven heteroplasmy could also involve species that hybridized and introgressed in the past and now are fully reproductively isolated. We tested this expectation, using as study-system two hydrenid beetle species belonging to the *Ochthebius* genus.

*Ochthebius quadricollis* and *O. urbanelliae* are distributed along the Mediterranean Sea coasts and occur syntopically in the same rock pools in a sympatric area along the coasts of the Italian peninsula^30,31^. They diverged during the Plio-Pleistocene^30,32^ as consequence of the climatic oscillations in the western Mediterranean region, that likely led to repeated isolation and secondary contact events between the two species^32^. Genetic analyses of sympatric populations using nuclear markers showed that hybridization and introgression occurred in the past, and then ceased due to the action of natural selection against maladaptive hybridization, which led to full reproductive isolation (speciation by reinforcement). No F1 hybrids between *O. quadricollis* and *O. urbanelliae* occur today in sympatric populations and the analysis of mating couples showed the occurrence of assortative mating. Furthermore, mating trials under laboratory conditions showed a pattern of higher premating isolation in sympatric versus allopatric populations^33^, and reproductive character displacement was found in sympatric populations of *O. urbanelliae*^34^.

Here, to furnish evidences to our hypothesis, we first searched for past mtDNA introgression, by using a phylogenetic approach; then, we screened *O. quadricollis* and *O. urbanelliae* individuals from sympatric and allopatric populations for the occurrence of mtDNA heteroplasmy, by developing a multiplex allele-specific PCR assay.

## Results

### mtDNA diversity and phylogenetic relationships

**Figure 1:**
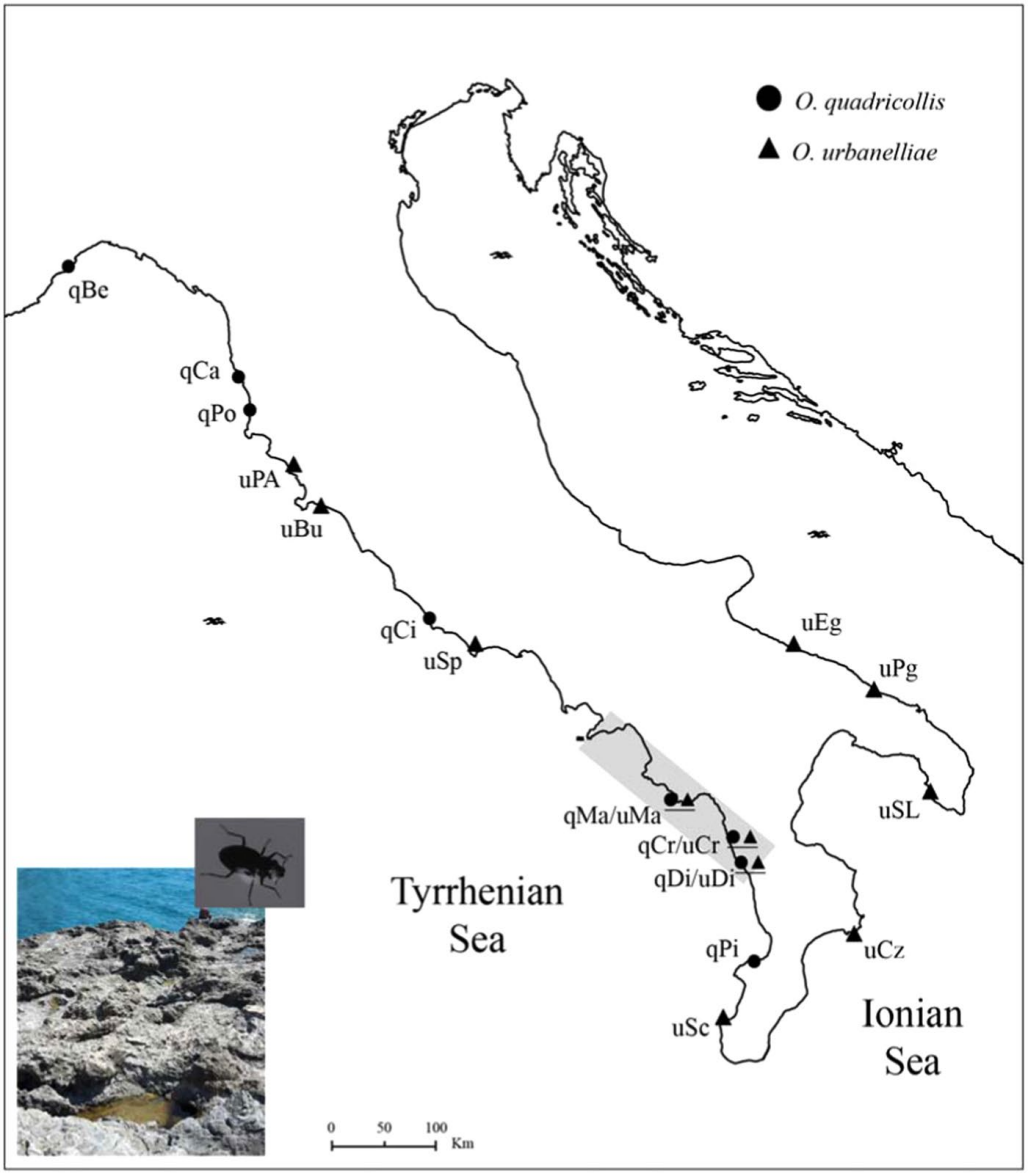
Map showing sampling sites of *Ochthebius quadricollis* and *O. urbanelliae* individuals. Light grey area: sympatric area between the two species (Urbanelli 2002). Photos show a specimen of *O. quadricollis* and a typical sea rock pool (Photo by Alessandra Spanò).

**Figure 2:**
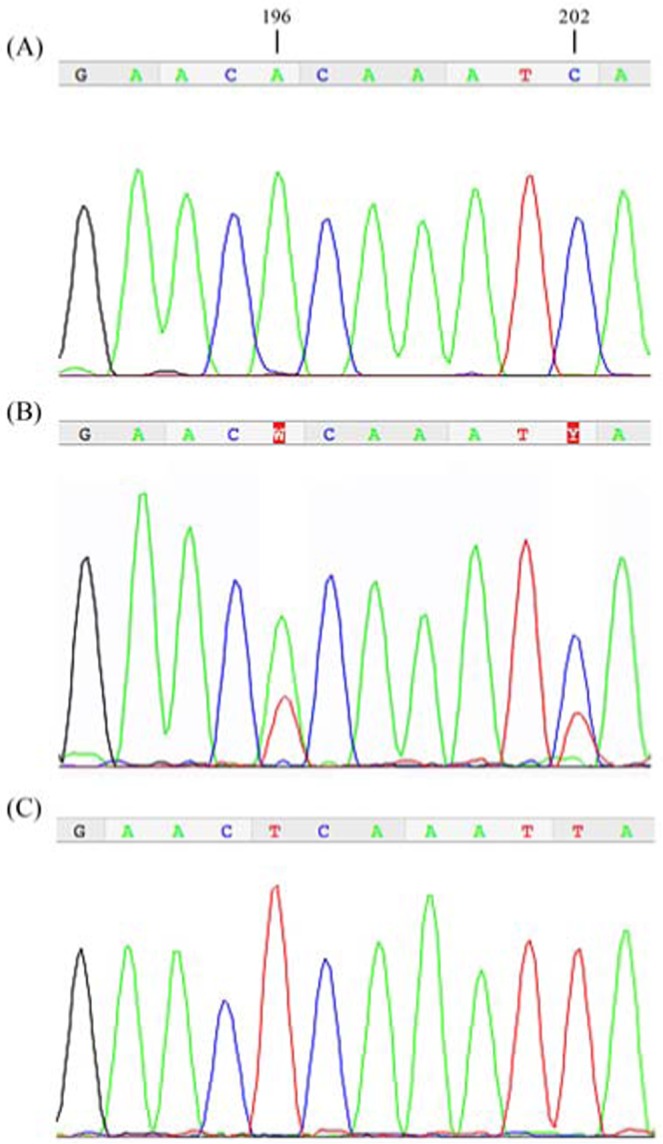
Chromatograms of the COI gene fragment showing two heteroplasmic positions. (**A**) *O. quadricollis* variant, (**B**) the sequence of the *O. urbanelliae* Cirella 3 heteroplasmic individual, (**C**) *O. urbanelliae* variant.

A total of 189 individuals (84 *O. quadricollis* and 105 *O. urbanelliae*) collected along the Italian coasts (Fig. 1, Table 1) were sequenced for a 537 base pair fragment in the 5′ half of the mitochondrial COI gene (Supplementary Fig. S1). No double peaks were observed in the sequence chromatograms of any individuals, with the exception of the *O. urbanelliae* Cirella-3 individual (Fig. 2). The obtained sequences showed a similarity of 99–100% with the sequences of *O. quadricollis* or *O. urbanelliae* available in GenBank. Two out of the 189 individuals were excluded from the following analyses because they were heteroplasmic (see below). The analysis of nucleotide and amino-acidic polymorphism detected 48 haplotypes (Supplementary Fig. S7), defined by 97 polymorphic sites and 111 mutations (106 synonymous) (Genbank accession number MH285878-MH285928). Average uncorrected *p*-distance between the two clades was 7.9%, while among populations it ranged from 0.006 to 0.032 in *O. quadricollis* and from 0 to 0.06 in *O. urbanelliae* (Supplementary Table S1).

**Table 1:** Sampled populations of *Ochthebius quadricollis* and *O. urbanelliae*.

Species	Locality	Latitude	Longitude	N	Haplotype	mtDNA introgressed individuals	Heteroplasmic individuals
*O. quadricollis*	Bergeggi	44.24°	8.42°	11	h1, h2, h3(2), h4(5), h5, h6	—	—
	Castiglioncello	43.40°	10.41°	11	h3(7), h7, h8, h9, h10	—	—
	Populonia	42.98°	10.98°	12	h3, h11(7), h12(2), h13(2)	—	—
	Circeo	41.22°	13.04°	10	h3, h14(7), h15, h16	—	—
	Cirella	39.71°	15.81°	12	h3, h11, h17(8), h34(2)	2	—
	Maratea	39.98°	15.70°	7	h17(4); h11, h13, h34	1	—
	Diamante	39.67°	15.81°	11	h6, h13(5), h17(4), h18	—	—
	Pizzo	38.73°	16.16°	10	h3, h5, h13(4), h19, h20(3)	—	—
*O. urbanelliae*	Punta Ala	42.80°	10.74°	10	h21(10)	—	—
	Burano	42.40°	11.38°	11	h21(10), h22	—	—
	Sperlonga	41.26°	13.42°	10	h23(6), h24(4)	—	—
	Maratea	39.98°	15.70°	12	h17, h25(2), h26, h27, h28, h29, h30, h31, h32, h33(2)	1	—
	Cirella	39.71°	15.81°	10	h34(5), h17(2), h35	3	2
	Diamante	39.67°	15.81°	—	—	—	—
	Scilla	38.24°	15.71°	11	h36(3), h37(7), h38	—	—
	Capo Rizzuto	38.90°	17.10°	10	h39(4), h40(2), h41(4)	—	—
	S. Maria Leuca	39.79°	18.75°	10	h42(7), h43(3)	—	—
	Pantanagianni	40.70°	17.84°	11	h44(6), h45(4), h46	—	—
	Egnazia	40.89°	17.37°	10	h44(5), h47(4), h48	—	—

The number of individuals analysed in each population and the haplotypes observed at mitochondrial CO I gene fragment are shown. In brackets is shown how many times each haplotype was found.

**Figure 3:**
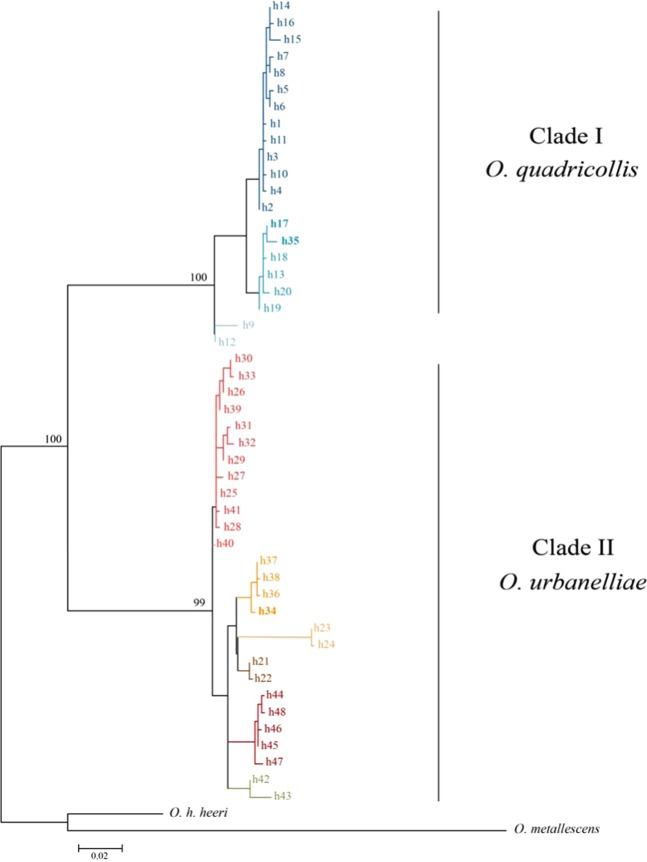
Phylogenetic relationships of the mtDNA haplotypes found in *Ochthebius quadricollis* and *O. urbanelliae*. Maximum likelihood (ML) tree is shown. Bootstrap values are shown above the main nodes. The haplotypes h17 and h34 (in bold) are shared between *O. quadricollis* and *O. urbanelliae* (Table 1); the haplotype h35 (in bold) was found in one *O. urbanelliae* individual (Table 1), but is more closely related to the *O. quadricollis* haplotypes.

Phylogenetic analyses using the Maximum Likelihood (ML) approach was performed to reconstruct the relationships among haplotypes (Fig. 3). All haplotypes of the clade I were found in *O. quadricollis* individuals with the exception of the haplotypes h17 that was found in both *O. quadricollis* and *O. urbanelliae* individuals, and with the exception of the haplotype h35 that was found in one *O. urbanelliae* individual (Fig. 3, Table 1). All haplotypes of the clade II were found in *O. urbanelliae* individuals with the exception of the h34 haplotype that was found in both *O. quadricollis* and *O. urbanelliae* individuals (Fig. 3, Table 1).

### Heteroplasmy

A multiplex allele-specific polymerase chain reaction (MAS-PCR) was used to investigate the occurrence of mtDNA heteroplasmy in *O. quadricollis* and *O. urbanelliae* (Supplementary Fig. S1). Both the MAS-PCR banding patterns and the sequencing results supported the specificity of the assay. Indeed, one band of the expected size was observed when the DNA of only *O. quadricollis* and *O. urbanelliae* was used, and two bands when DNA was mixed (Supplementary Fig. S2). Likewise, the sequences of the amplicons showed 100% identity with *O. quadricollis* and *O. urbanelliae* sequences. The MAS-PCR assay also proved to be sensitive, being able to detect an amount as low as 0.01 ng of *O. quadricollis* mtDNA (ratio *O. urbanelliae*/*O. quadricollis* mtDNA, 1:500), and an amount as low as 0.005 ng of *O. urbanelliae* mtDNA (ratio *O. quadricollis*/*O. urbanelliae* mtDNA, 1:1000) (Supplementary Fig. S2).

The MAS-PCR assay was then used to analyze all individuals that were sequenced for the COI mtDNA gene fragment to check for mitochondrial heteroplasmy. In *O. urbanelliae*, two heteroplasmic individuals were found (i.e. one male and one female) (Table 1). They showed two electrophoretic bands following MAS-PCR (Supplementary Fig. S3), which sequences were identical to a fragment of the *O. quadricollis* and *O. urbanelliae* haplotypes. In *O. quadricollis*, no heteroplasmic individuals were found.

## Discussion

Paternal leakage and mtDNA heteroplasmy have been documented in several animal species, including insects, fishes, reptiles, birds and mammals^8,10^. Being more difficult to detect when mtDNA variation within or among populations is low, these phenomena have been often observed in secondary contact zones or laboratory crosses, as a consequence of hybridization between heterospecific individuals or between homospecific individuals belonging to highly divergent genetic lineages^8,10,35^. Here, we hypothesized that hybridization can lead to mtDNA paternal leakage and heteroplasmy also via mtDNA introgression. By using genetic data, we showed that: *i*) mtDNA introgression occurred between the two species and that mtDNA-introgressed and pure individuals co-occur in natural populations; *ii*) heteroplasmic individuals are present in *O. urbanelliae*; *iii*) mtDNA heteroplasmy originated through paternal leakage.

First, the phylogenetic analyses showed that *O. quadricollis* and *O. urbanelliae* share mtDNA haplotypes, as well as having some haplotypes more closely related to those of the other species than to those of its own haplogroup (Fig. 3, Table 1). This pattern is consistent with a process of mtDNA introgression and it is concordant with the signatures in the nuclear genomes of introgressive hybridization between *O. quadricollis* and *O. urbanelliae*, before the completion of the speciation process by reinforcement, shown by nuclear markers^31,33^. Furthermore, alternative hypotheses, such as incomplete lineage sorting can be excluded^20^. Contrary to what would be expected under this hypothesis, the introgressed haplotypes between *O. quadricollis* and *O. urbanelliae* are indeed not randomly distributed across the species’ range, but rather they are geographically localized within the sympatric area between the two species, where both mtDNA-introgressed and pure individuals co-occur (Fig. 1, Table 1).

Second, the screening of *O. quadricollis* and *O. urbanelliae* individuals by MAS-PCR and sequence analyses showed the occurrence of heteroplasmic individuals in *O. urbanelliae* (Table 1, Supplementary Fig. S3). Misleading results due to unspecific PCR amplification of the mtDNA of the two species can be confidently ruled out, as the MAS-PCR assay proved to be highly specific. Furthermore, the sequences obtained from the amplicons were identical to the COI sequences of *O. quadricollis* and *O. urbanelliae* found in the sympatric area, which also exclude the possibility that non-functional nuclear copies of mitochondrial genes (NUMTs) have been analysed.

Third, mtDNA heteroplasmy originated by paternal leakage. Empirical evidence showed that different routes can lead to mtDNA heteroplasmic individuals^8,10^. When mtDNA heteroplasmy is due to the leakage of paternal mitochondrial genome, it is expected that the mtDNA haplotypes within the heteroplasmic individuals derive from both maternal and paternal mtDNA lineages^8,10,14^. The heteroplasmic individuals found in *O. urbanelliae* are concordant with this expectation, as they showed one haplotype identical to the haplotypes of *O. quadricollis*, and the other identical to those of *O. urbanelliae*, which supports the hypothesis that they originated by paternal leakage. In *O. quadricollis*, no heteroplasmic individuals were found by the MAS-PCR screening (Table 1). Genetic or selective factors preventing paternal leakage in this species could be invoked to explain this result, or alternatively, heteroplasmic individuals may have escaped our screening by chance, if *O. urbanelliae* DNA was below our detection limit.

Taking into account the above results and the reproductive isolation among the two species, we argue that mtDNA introgression promoted paternal leakage in the *Ochthebius* beetles. Events of paternal leakage and heteroplasmy have often been ascribed to a failure of the recognition mechanisms between nuclear and mitochondrial genomes located in the eggs and sperms, and often they have been shown to involve hybridization between divergent lineages^8,36^. However, hybridization between *O. quadricollis* and *O. urbanelliae* cannot be invoked to explain paternal leakage and mtDNA heteroplasmy in these species, because reproductive isolation between them is completed^31,33^. Likewise, the hypothesis that paternal leakage originated from past hybridization events between *O. quadricollis* and *O. urbanelliae* and then was followed by maternal transmission of heteroplasmy can be excluded as well, because the heteroplasmic variants would have disappeared over time since the completion of reproductive isolation between the two species^30,37–39^. Paternal leakage and mtDNA heteroplasmy in the *O. quadricollis* and *O. urbanelliae* beetles would therefore be due to mates between mtDNA-introgressed and pure conspecific individuals.

The role of the mtDNA introgression as a source of evolutionary novelty has become increasingly recognized in recent years^40–43^. Our results, by showing that mtDNA introgression can promote paternal leakage, highlight that introgression would be able to generate genetic novelty in a way that has been neglected to date. Furthermore, they support that the frequency and distribution of paternal leakage and mtDNA heteroplasmy can be deeply underestimated in natural populations, as the commonly used PCR-Sanger sequencing approach can fail to detect mitochondrial heteroplasmy, and specific studies aimed at searching for heteroplasmy in populations where mtDNA-introgressed and pure individuals co-occur remain scarce.

In animals, the mtDNA genome has recently been shown also to recombine, both at intra- and intermolecular level^8,14,44^, and recombination has been suggested as an important evolutionary mechanism avoiding deleterious mutation meltdown in mtDNA (i.e. the Muller’s ratchet), as well as for the origin of new genetic combinations. Paternal leakage and mtDNA heteroplasmy, allowing two different genomes to meet, represent an intermediate but critical step for recombination. An understanding of how frequently they occur in nature, as well as the processes underlying them, represent therefore outstanding questions to be addressed.

## Materials and Methods

### Samples and species recognition

*Ochthebius quadricollis* and *O. urbanelliae* adult individuals were collected from 16 localities along the Italian coasts (Table 1, Fig. 1). All individuals were identified using the morphological and biochemical keys of Audisio *et al*.^45^ and Urbanelli *et al*.^30^. Standard horizontal starch gel electrophoresis was performed following the protocols described in Urbanelli^31^.

### mtDNA sequencing and phylogenetic inferences

DNA from single adults was extracted from the tissue homogenate used for allozymic analyses by standard CTAB (cetyltrimethyl ammonium bromide) protocol^46^. Partial sequences of the Cytochrome Oxidase I (COI) mitochondrial gene were obtained through PCR-amplification. The universal primers pair C1-J-2183 and TL2-N-3014 was used to amplify and sequencing a COI fragment of ~900 base pairs^47,48^. The following specific primers were then designed and used for further analyses: *Ocht*COI-f 5′-accaggatttggaataattt-3′ and *Ocht*COI-r 5′-tccaatagaagaaataatatttc-3′. The PCR was carried out in a 25 µl volume containing 5 ng of DNA, 10 mMTris-HCl, pH 8.3, 2.0 mM MgCl_2_, 0.2 μM of the forward and reverse primers, 0.4 mM dNTPs, 0.3 units of NZYTaq polymerase (NZYtech, Lisbon, Portugal) and water. Negative controls including all reagents but DNA were also included in the reaction. PCR cycling procedure was: 95 °C for 5 min followed by 34 cycles of 93 °C for 1 min, 55 °C for 1 min, 72 °C for 1 min 30 s, and a single final step at 72 °C for 10 min. PCR sequences were obtained using an ABI PRISM 3700 DNA sequencer by GATC Inc. (www.GATC.com). All individuals were double strand sequenced. The individuals found introgressed were amplified multiple times and two independent PCR products were sequenced.

Sequences were edited and aligned using the software Chromas 2.6.5 (Technelysium, Helensvale, Australia) and ClustalX 2.1^49^, respectively, and compared with the *O. quadricollis* and *O. urbanelliae* sequences available in GenBank using BLASTN algorithm. Nucleotide and amino-acidic polymorphisms of the COI gene fragments were estimated using the software DNAsp 6^50^. The average uncorrected *p*-distance between groups of haplotypes and populations was computed using Mega 7.0^51^.

Phylogenetic relationships among haplotypes of *O. quadricollis* and *O. urbanelliae* were inferred using the Maximum Likelihood (ML) method, as implemented in PAUP 4.0^52^. ML analysis was performed using heuristic searches with 100 rounds of random sequence addition and tree bisection-reconnection (TBR) branch swapping algorithm. The GTR + G + I substitution model was used (gamma shape parameter G = 1.480; proportion of invariable sites I = 0.669), following the substitution model inferred by jModelTest 2.0^53^ using the Akaike Information Criterion. The robustness of the inferred ML tree topology was assessed by the non-parametric bootstrap method with 1000 replicates. *Ochthebius heeri heeri* (KT804242.1) and *Ochthebius metallescens* (HF931191.1) were used as outgroups in the phylogenetic analyses^54^.

### Heteroplasmy screening

To screen the *Ochthebius* individuals for mtDNA heteroplasmy, a multiplex allele-specific polymerase chain reaction (MAS-PCR) was designed. On the basis of the CO I sequences obtained from the *O. quadricollis* and *O. urbanelliae* individuals, a common forward primer for the two species (*OchCom_F*, 5′-gcggtattttaagcttttca-3′) and a specific reverse primer for *O. quadricollis* (*OchQua_R*, 5′-cttgattcaaattgacatc-3′) and *O. urbanelliae* (*OchUrb_R*, 5′-actgcaccttgacttaatataa-3′) were designed. The common primer and the two species-specific primers were combined into a single multiplex PCR reaction to yield distinct PCR banding patterns that can accurately detect the mtDNA of the two species (Supplementary Fig. S1).

The specificity of the assay (i.e. the ability to correctly discriminate the mtDNA of the two species), was tested by amplifying and sequencing the DNA of known *O. quadricollis* and *O. urbanelliae* individuals and a mixture of the two species. PCR reactions were carried out in a 25 µl volume containing 5 ng of DNA, 10 mMTris-HCl, pH 8.3, 2.0 mM MgCl_2_, 0.4 mM dNTPs, 0.4 µM of the common forward primer, 0.4 µM of each species-specific reverse primer, and 2.5 units of NZYTaq polymerase (NZYtech, Lisbon, Portugal). In each reaction, negative controls containing all reagents but DNA were also included. PCR cycling procedure was: 95 °C for 5 min followed by 34 cycles at 93 °C for 1 min, 55 °C for 1 min, 72 °C for 1 min 30 s, and a single final step at 72 °C for 10 min. The PCR products were then separated by electrophoresis run on 2% agarose, 0.5X TAE gel, and visualized by staining with Gelred (Sigma-Aldrich, Milan, Italy). The sizes of the DNA fragments were assessed using the 100 bp DNA ladder (Promega, Milan, Italy) run on the same gel. Three technical replicates of each reaction were performed to check for the reproducibility of the MAS-PCR assay. PCR products obtained from the amplification of *O. quadricollis* and *O. urbanelliae* individuals and mixed DNA samples were double strand sequenced (https://www.gatc-biotech.com). The bands of the mixed DNA samples were excised from the gel, purified using the NucleoSpin gel and the PCR Clean-up purification kits (Macherey-Nagel, Düren, Germany) and then double strand sequenced.

The sensitivity of the MAS-PCR assay (i.e. the smallest amount of mtDNA of both species in a sample that can be accurately detected), was tested by amplifying the DNA of *O. quadricollis* and *O. urbanelliae* at different ratios. The amount of *O. quadricollis* (or *O. urbanelliae*) DNA was gradually reduced to 0.5, 0.05, 0.01 and 0.005 ng to obtain dilutions of *O. quadricollis* to *O. urbanelliae* DNA at 1:10, 1:100, 1:500 and 1:1000. Multiplex PCR amplifications were performed and PCR products were separated by electrophoresis as described above.

After the specificity and sensitivity tests, the MAS-PCR assay was used to screen for heteroplasmy the sampled *O. quadricollis* and *O. urbanelliae* individuals. The heteroplasmic individuals were amplified three times to check for consistency, and the amplicons were excised from the gel, purified and double strand sequenced, as described above.

## Supplementary information

Supplementary information

## Data Availability

All data generated or analysed during this study are included in this published article
