# Supplementary material for: Ancient hybridization and mtDNA introgression behind current paternal leakage and heteroplasmy in hybrid zones
Source: Sci Rep. 2019 Dec 16;9:19177. doi: 10.1038/s41598-019-55764-w (PMC6914795; doi:10.1038/s41598-019-55764-w)
Supplement: Supplementary file 1 — Supplementary information [file 41598_2019_55764_MOESM1_ESM.pdf]

Ancient hybridization and mtDNA introgression behind current paternal leakage and heteroplasmy in hybrid zones

Valentina Mastrantonio[1], Sandra Urbanelli[1], Daniele Porretta[1*]

[1] Department of Environmental Biology, Sapienza University of Rome, Rome, Italy

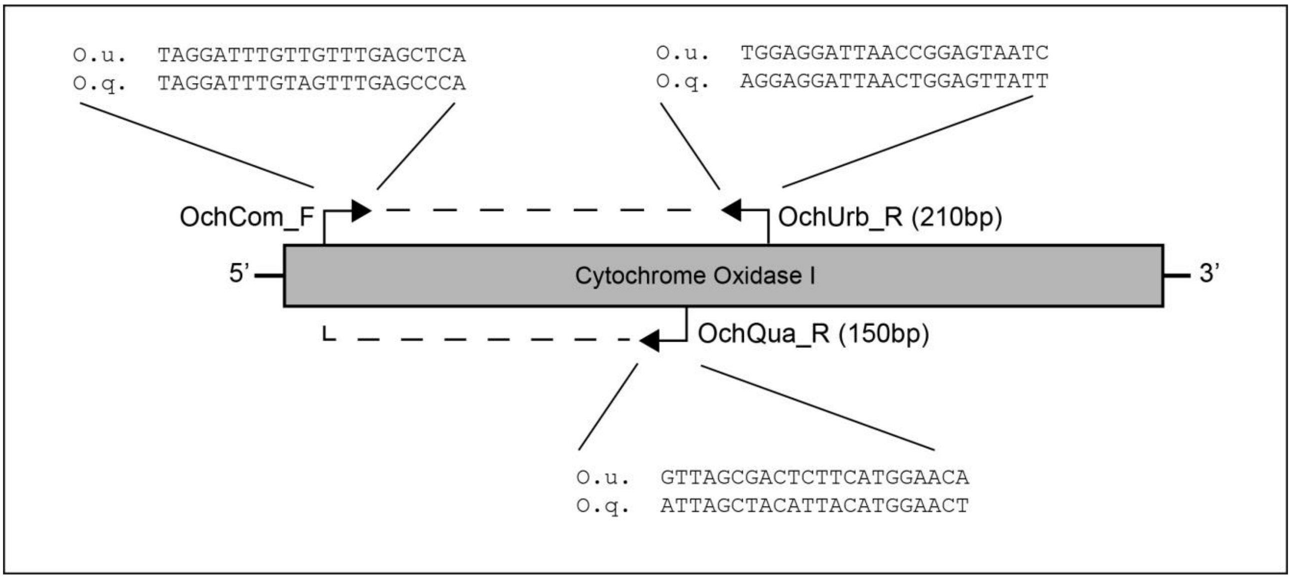

Figure S1. Schematic representation of the primer pairs used in the MAS-PCR assay. Primer pair OchCom_F/ OchUrb_R: PCR product of 210 bp; Primer pair OchCom_F/ OchQua_R: PCR product of 150 bp.

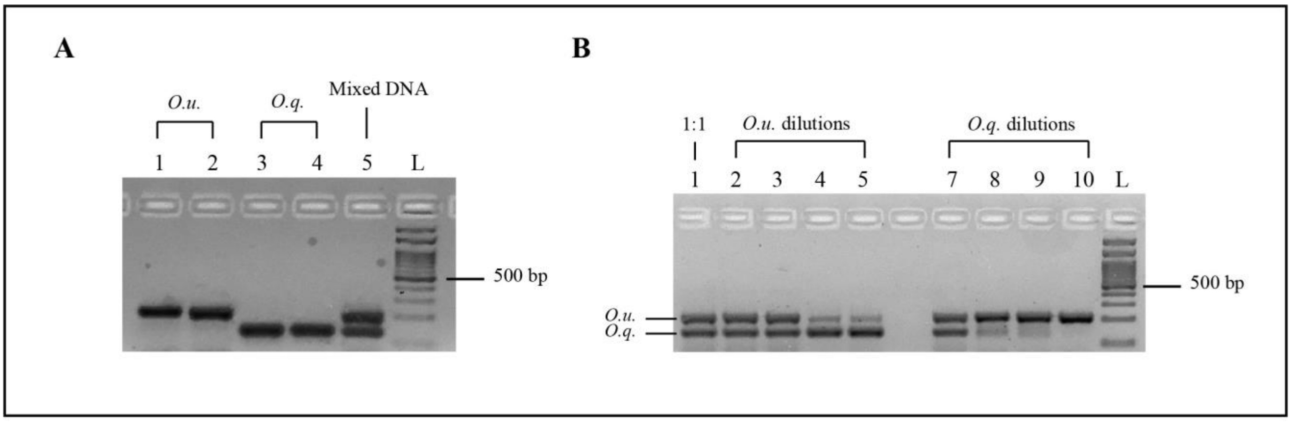

Figure S2. Specificity and sensibility tests of the MAS-PCR assay**.** (A) *Specificity test.* Electrophoretic pattern of known *Ochthebius urbanelliae* DNA (lanes 1-2), *O. quadricollis* DNA (lanes 3-4) and mixed DNA (*O.u.* + *O.q.*) (lane 5). Lane L: 100 bp DNA ladder. (B) *Sensitivity test.* Electrophoretic pattern of mixed DNA between *O. urbanelliae* and *O. quadricollis* diluted at different ratios. Starting DNA concentration 5 ng/µl the DNA of one of the two species was gradually reduced. Lanes 1: (1:1) dilution; lines 2-5: *O.u.* dilutions (1:10), (1:100), (1:500), (1:1.000); Lanes 7-10: *O.q.* dilutions (1:10), (1:100), (1:500), (1:1.000); Lane L: 100 bp DNA ladder. Upper band: *O. urbanelliae* (*O.u.*); lower band: *O. quadricollis* (*O.q.*). The original photos of the gels are shown in the Supplementary figures S4 and S5.

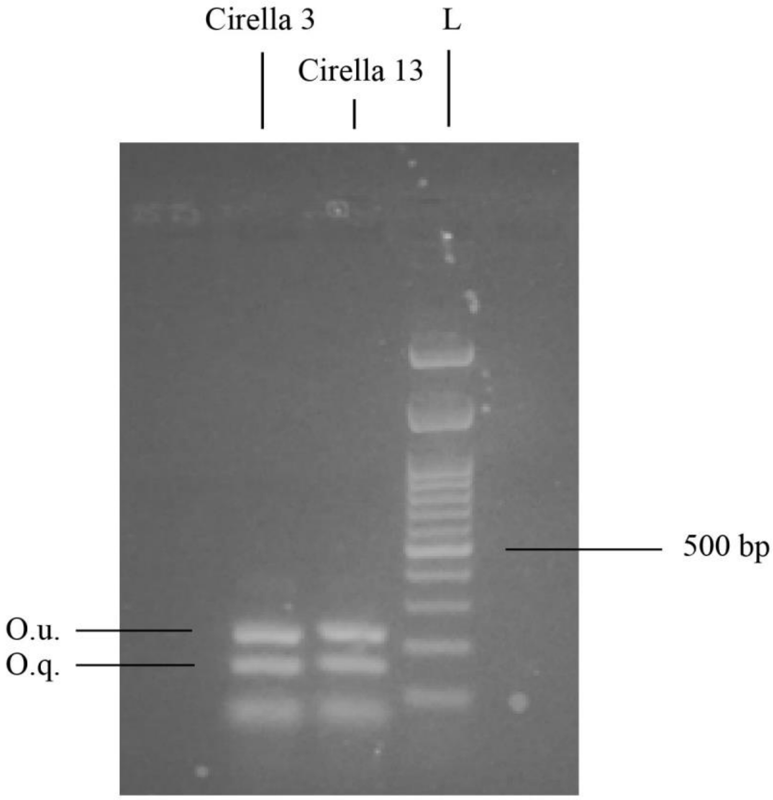

Figure S3. Electrophoretic pattern of the heteroplasmic *Ochthebius urbanelliae* individuals following MAS-PCR (lines 1 and 2). Upper band: *O. urbanelliae* (*O.u.*); lower band: *O. quadricollis* (*O.q.*). L: DNA ladder 100 bp. The original photos of the gels are shown in the Supplementary figure S6.

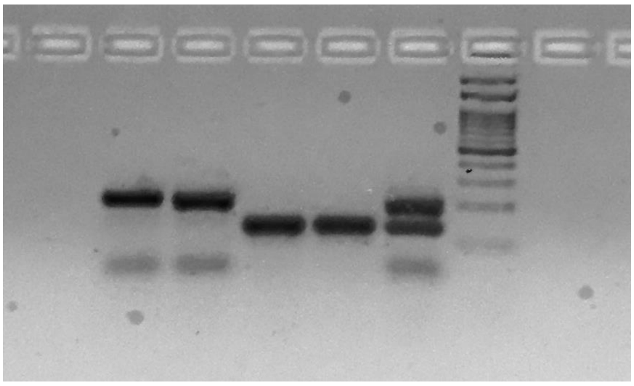

Figure S4. Original photo of the electrophoretic gel showed in Figure S2A.

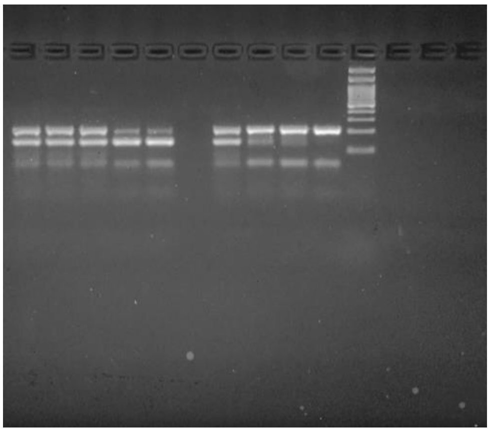

Figure S5. Original photo of the electrophoretic gel showed in Figure S2B.

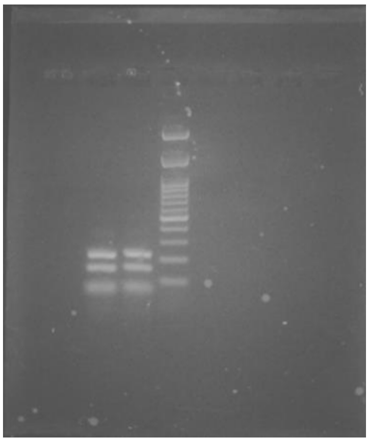

Figure S6. Original photo of the electrophoretic gel showed in Figure S3.

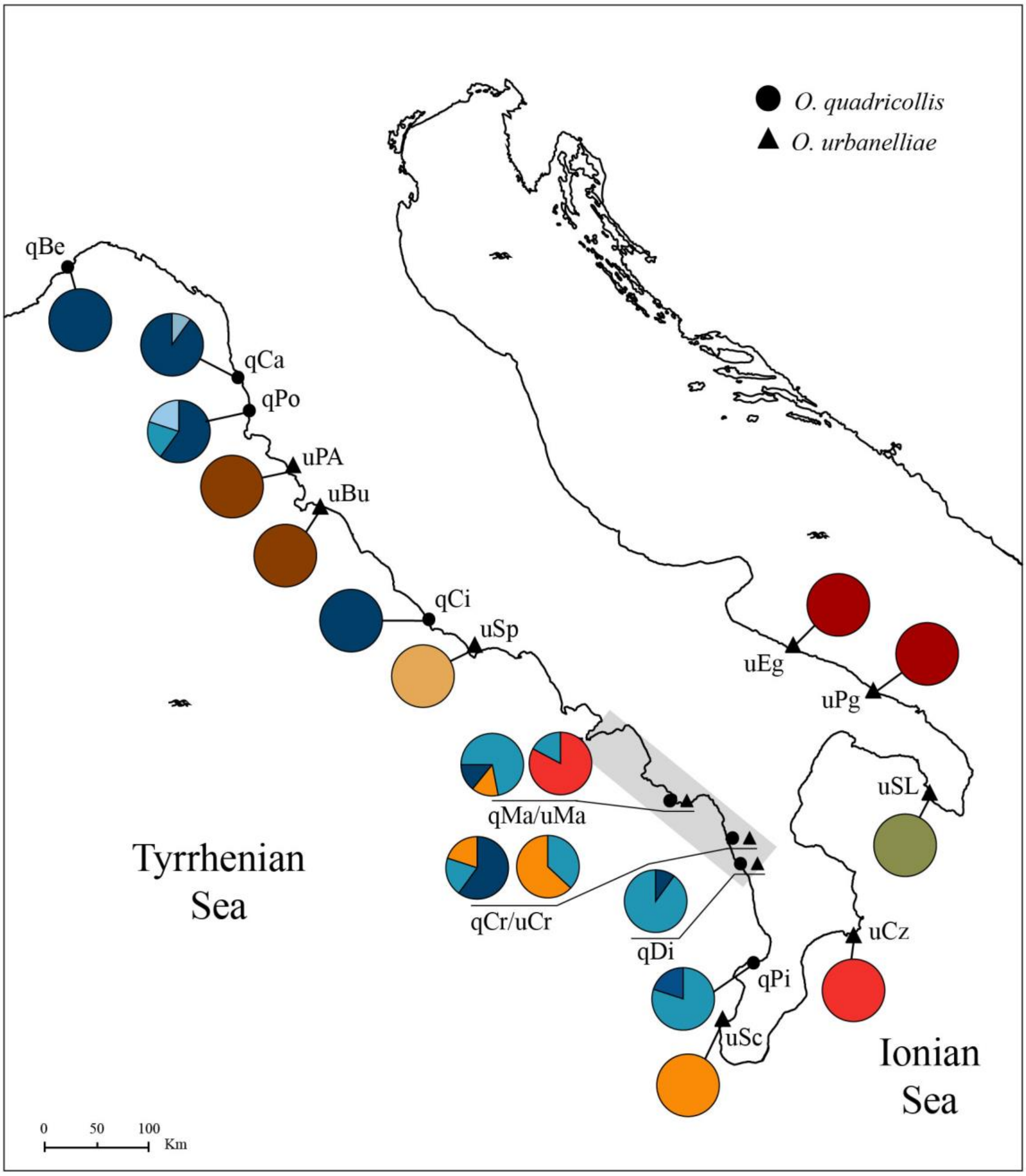

Figure S7. Geographic distirbution of haplotypes in the *O. quadricollis* and *O. urbanelliae* populations. Haplotypes are coloured according to Figure 3 in the paper text.

Table S1. Average uncorrected p-distance among the *Ochthebius quadricollis* and *O. urbanelliae* populations analysed. Populations are encoded as in the Table 1 of the paper.

	qBe	qCa	qPo	qCi	qCr	qMa	qDi	qPi	uPa	uBu	uSp	uMa	uCi	uSc	uCz	uSL	uPg	uEg
qBe	****																	
qCa	0.006	****																
qPo	0.009	0.010	****															
qCi	0.006	0.007	0.011	****														
qCr	0.029	0.030	0.029	0.031	****													
qMa	0.028	0.029	0.027	0.029	0.032	****												
qDi	0.016	0.017	0.015	0.018	0.023	0.020	****											
qPi	0.016	0.017	0.016	0.019	0.025	0.022	0.007	****										
uPa	0.102	0.101	0.099	0.099	0.088	0.090	0.102	0.102	****									
uBu	0.102	0.101	0.099	0.099	0.088	0.090	0.102	0.102	0.000	****								
uSp	0.107	0.106	0.105	0.106	0.096	0.098	0.111	0.110	0.023	0.023	****							
uMa	0.083	0.083	0.083	0.081	0.077	0.078	0.086	0.086	0.031	0.031	0.040	****						
uCi	0.073	0.073	0.071	0.072	0.065	0.066	0.070	0.071	0.050	0.050	0.060	0.054	****					
uSc	0.109	0.108	0.107	0.106	0.094	0.097	0.113	0.112	0.016	0.016	0.031	0.036	0.047	****				
uCz	0.090	0.090	0.090	0.088	0.082	0.084	0.094	0.094	0.022	0.022	0.032	0.014	0.051	0.026	****			
uSL	0.097	0.096	0.097	0.095	0.089	0.091	0.101	0.101	0.021	0.021	0.033	0.034	0.056	0.030	0.025	****		
uPg	0.101	0.100	0.101	0.098	0.091	0.093	0.105	0.104	0.024	0.023	0.036	0.035	0.057	0.030	0.026	0.026	****	
uEg	0.101	0.099	0.100	0.098	0.091	0.093	0.104	0.104	0.024	0.024	0.038	0.036	0.058	0.031	0.027	0.025	0.004	****
